# Elucidation of Brain Morphogenesis Using Quantitative Brain Magnetic Resonance Imaging in Children

**DOI:** 10.1111/ped.70450

**Published:** 2026-06-21

**Authors:** Tadashi Shiohama

**Affiliations:** ^1^ Department of Pediatrics, Graduate School of Medicine Chiba University Chiba Japan; ^2^ Department of Pediatrics International University of Health and Welfare Narita Hospital Chiba Japan

**Keywords:** autism spectrum disorder, brain morphology, diffusion magnetic resonance imaging, diffusion tensor imaging, harmonization

## Abstract

Quantitative brain magnetic resonance imaging has revolutionized pediatric neurodevelopment research by enabling noninvasive, reproducible, and high‐resolution assessments of brain morphology across the entire brain. Advances in anatomical structure analysis and diffusion‐weighted tractography now permit detailed characterization of gray and white matter, cortical thickness, surface area, gyrification, and fiber integrity throughout development. Automated processing pipelines, including FreeSurfer, FSL, and CIVET, have supported large‐scale analyses, while harmonization frameworks and normative growth curves have facilitated clinical translation. Diffusion tensor imaging (DTI) provides complementary insights into white matter microstructure, revealing neurodevelopmental trajectories and disorder‐specific connectivity alterations. These approaches have identified structural biomarkers in multiple conditions, including reduced nucleus accumbens volume and ventricular enlargement in autism spectrum disorder (ASD), as well as early amygdala overgrowth and glymphatic dysfunction that may predict ASD onset. Despite these advances, several challenges remain, such as inter‐scanner variability, age‐dependent processing limitations, and the lack of validated individual‐level biomarkers. Standardization of imaging protocols and robust statistical harmonization will be essential to overcome these obstacles and enable longitudinal, patient‐specific assessments. The incorporation of quantitative magnetic resonance imaging into clinical workflows holds promise for early diagnosis, individualized monitoring, and therapeutic stratification of neurodevelopmental and genetic disorders. Ultimately, comprehensive morphometric and diffusion‐based profiling will advance understanding of brain morphogenesis and drive precision medicine in pediatric neurology.

## 1 Introduction

The assessment of brain morphology historically began as comparative anatomy aimed at evaluating differences among interspecies and interethnic groups. Advances in neuroimaging techniques have since expanded the research scope to include individuals with psychological and genetic disorders. Brain magnetic resonance imaging (MRI) is the most widely used neuroimaging technique in both clinical and research settings because it enables noninvasive, highly reproducible, and high‐resolution evaluation of the entire brain. In addition to identifying structural characteristics, pathological origins—such as ischemia, hemorrhage, inflammation, and demyelination—can be inferred from signal intensities across multiple sequences and from the timing of brain insults. Although various structural and signal abnormalities have been detected through qualitative approaches, the inter‐ and intra‐observer reproducibility of cerebral atrophy assessment remains extremely low [1, 2].

Since the clinical adoption of brain MRI in the 2000s, the main focus of morphological analysis has been qualitative or semi‐quantitative assessment using manual tracing methods [3, 4]. Although traditional structural MRI brain provides valuable anatomical information, it lacks sensitivity in detecting subtle deviations from normal neurodevelopmental trajectories. The emergence of modern high‐field scanners with advanced sequence settings has enabled quantitative evaluation through automated tracing methods.

Importantly, the human brain's structural organization is closely linked to its functional capacity, although it is often unclear whether structural changes cause or result from brain dysfunction. For example, a meta‐analysis reported that total intellectual quotient scores in adults were positively associated with whole‐brain volume [5] and regional cortical thickness [6, 7]. Similarly, MRI‐based morphological studies have identified distinct structural patterns in patients with psychological, mood, and neurodevelopmental disorders—such as autism spectrum disorder and attention‐deficit/hyperactivity disorder—compared with healthy controls; this approach has also been applied to genetic disorders. Several quantitative methods are used to analyze the brain, including anatomical structure analysis with three‐dimensional T1‐weighted imaging [8, 9, 10, 11, 12, 13], white matter fiber analysis using diffusion‐weighted imaging [14, 15, 16, 17], proton magnetic resonance spectroscopy (^1^H‐MRS) [18, 19], arterial spin labelling (ASL) [20], multiparametric MRI [21], MR fingerprinting [22], and functional MRI (fMRI) [23]. This mini‐review primarily focuses on anatomical structure analysis and white matter fiber analysis.

## 2 Evaluation by the Anatomical Structure Analysis

Brain development from the fetal period through adolescence involves dynamic changes in cortical thickness, volume, surface area, and myelination [24, 25, 26]. Volumetric expansion of cortical gray matter (CGM) and white matter (WM) is most prominent during the first 2 years after birth, with CGM showing gradual increases throughout childhood and WM continuing to grow until approximately 30 years of age [27, 28]. Anatomical structural analyses are typically performed using three‐dimensional T1‐weighted images. Quantitative MRI methodologies, such as voxel‐based morphometry (VBM) and surface‐based morphometry (SBM), provide comprehensive morphometric evaluations across the entire brain [29, 30, 31]. VBM allows for the measurement of brain tissue volumes, including CGM, WM, subcortical regions, and ventricles. In contrast, although SBM does not assess subcortical regions, it enables the analysis of cortical thickness, surface area, and curvature using standardized atlases such as the Desikan–Killiany atlas [32], the Destrieux atlas [33], and the DKT atlas [34]. Furthermore, anatomical structure analysis allows for the calculation of various indices that represent the degree of gyral and sulcal formation at each local site. Depending on the computational method, indices such as the local gyrification index [35], folding index [36], intrinsic curvature index [37], mean curvature [37], and Gaussian curvature [37] can be derived. Although immature gyrus and sulcus formation are believed to influence cortical function in the corresponding regions and the organization of nerve fibers originating there [38], the morphology of the gyri and sulci is shaped by complex factors. Therefore, even when characteristic changes in these indices are detected, their pathophysiological interpretations remain challenging.

**FIGURE 1:**
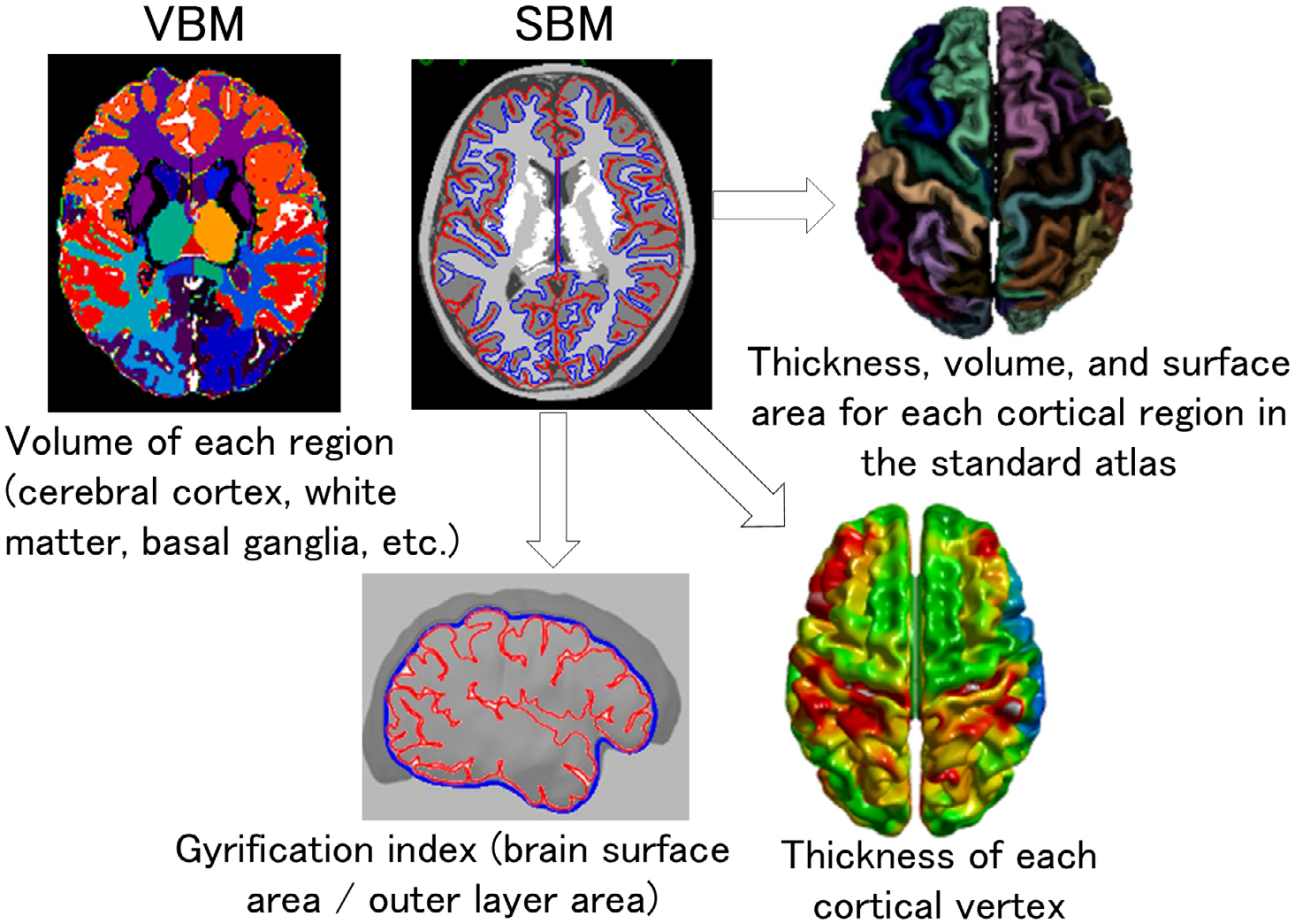
Quantitative MRI analysis according to voxel‐based morphometry (VBM) and surface‐based morphometry (SBM) using the CIVET pipeline [9].

For the comprehensive brain morphometry using these methods, several established automated programs—such as FreeSurfer (https://surfer.nmr.mgh.harvard.edu/) [8], CIVET (https://mcin.ca/technology/civet/) (Figure 1) [9], FMRIB Software Library (FSL) (http://fsl.fmrib.ox.ac.uk/fsl/fslwiki/) [10], and Statistical Parametric Mapping (SPM) (http://www.fil.ion.ucl.ac.uk/spm/) [11]—provide reproducible outputs across studies and users. Although some of these tools overlap in functionality, several web‐based platforms, including cBrain (https://cbrain.ca/) [39], Brainlife.io (https://brainlife.io/) [40], volBrain (https://volbrain.net/) [12], and MRIcloud (https://mricloud.org/) [13], also support comprehensive neuroimaging analyses.

As genomic diagnostics become increasingly prevalent, deep phenotyping via neuroimaging—an approach known as next‐generation phenotyping—provides an opportunity to redefine clinical spectra [41]. Comprehensive brain morphology analysis using MRI enables consistent evaluation of patients with genetic abnormalities and their corresponding rodent models within a single modality, making it a valuable tool for elucidating disease mechanisms and assessing drug efficacy. However, when elucidating gene functions in brain morphogenesis using MRI, particular caution is required when interpreting regions where anatomical structures differ between humans and rodents, such as the temporal sulcus and cortical gyri [42].

## 3 Current Limitations and Overcoming Them in the Anatomical Structure Analysis

Despite significant methodological advances, several challenges remain before automated analysis programs can be widely adopted for clinical image diagnosis. Long processing times (e.g., 4–5 h per scan in FreeSurfer), interscanner variability, and poor image quality can limit their utility in clinical settings [43, 44]. Although visual inspection of the segmentation results and occasional manual correction of the pial surface or voxel boundaries are often required, manual intervention does not necessarily improve regional segmentation quality [45, 46]. Moreover, most normative datasets for standard atlases have been derived from Western populations, raising concerns regarding cross‐population generalizability and underscoring the need for region‐specific calibration.

Early childhood imaging presents unique challenges, including rapid myelination, low gray–white contrast, and motion artifacts that increase failure rates, thereby necessitating further optimization of infant‐specific processing pipelines [47, 48, 49]. With high‐quality input images and careful quality control, the adult FreeSurfer pipeline can process images of children aged 4.5 years and older [50], whereas Infant FreeSurfer [47] was originally designed for images of children younger than 2 years. The latest Infant FreeSurfer has been merged into the main branch of FreeSurfer (version 8.1), extending its applicability to the 2–5 year age range. Similarly, iBEAT v2.0 provides a streamlined, deep learning–based pipeline tailored for ages 0–6 years, offering robust skull stripping, tissue segmentation, and surface extraction [48]. M‐CRIB‐S, updated for neonatal and infant surface‐based analysis compatible with FreeSurfer conventions, further improves anatomical accuracy in this population [49]. However, the absence of a unified image‐processing pipeline spanning infancy, childhood, and adulthood represents a critical limitation for longitudinal neuroimaging. Because different pipelines are optimized for specific developmental stages—such as Infant FreeSurfer for early life and standard FreeSurfer for older individuals—systematic differences in segmentation algorithms, tissue contrast handling, and atlas definitions may introduce artificial discontinuities across time points [47, 48]. These methodological inconsistencies can obscure true biological trajectories and may lead to erroneous interpretations of developmental change, particularly in studies aiming to detect subtle deviations associated with neurodevelopmental disorders.

From a clinical perspective, this limitation poses a significant barrier to real‐world implementation, where continuous monitoring of individual patients over time is essential. Without harmonized processing across age ranges, apparent changes in brain metrics may reflect pipeline‐related artifacts rather than true disease progression or treatment response [51]. Therefore, the development of unified, lifespan‐compatible processing frameworks and cross‐age harmonization strategies is crucial for enabling reliable longitudinal assessment and clinical translation [52, 53].

Another challenge lies in the difficulty of evaluating brain morphological features in individual cases, even though group‐level differences can be readily detected. The widespread clinical adoption of morphometric analysis is hindered by three main factors: inter‐scanner variability, a lack of age‐ and sex‐specific normative datasets, and the absence of validated disorder‐specific biomarkers. Differences in scanner hardware and imaging protocols can introduce artificial variances in morphometric measurements. Although differences in whole‐brain volume between field strengths are generally minimal and acceptable for comparative analyses [54], other reports have indicated scanner‐induced changes up to 0.59 standard deviations in gray matter volume [44] and approximately 0.4 mm in cortical thickness [43] among healthy adults.

To control intermachine bias, several methods have been employed, including illustrating the effects of differences between scanners [55], using quantitative evaluations with MRI phantoms that simulate biological tissue [56], and imaging the same participants across multiple scanners (traveling subjects) [57]. In recent studies, statistically based harmonization techniques such as ComBat methodologies [58, 59, 60] have also been applied. Tools such as ComBat and ComBat‐GAM statistically remove batch effects while preserving biological signals [58, 59]. The newer ComBatLS additionally maintains variance structure, mitigating issues that arise when demographic or disease status distributions differ across sites [60].

**FIGURE 2:**
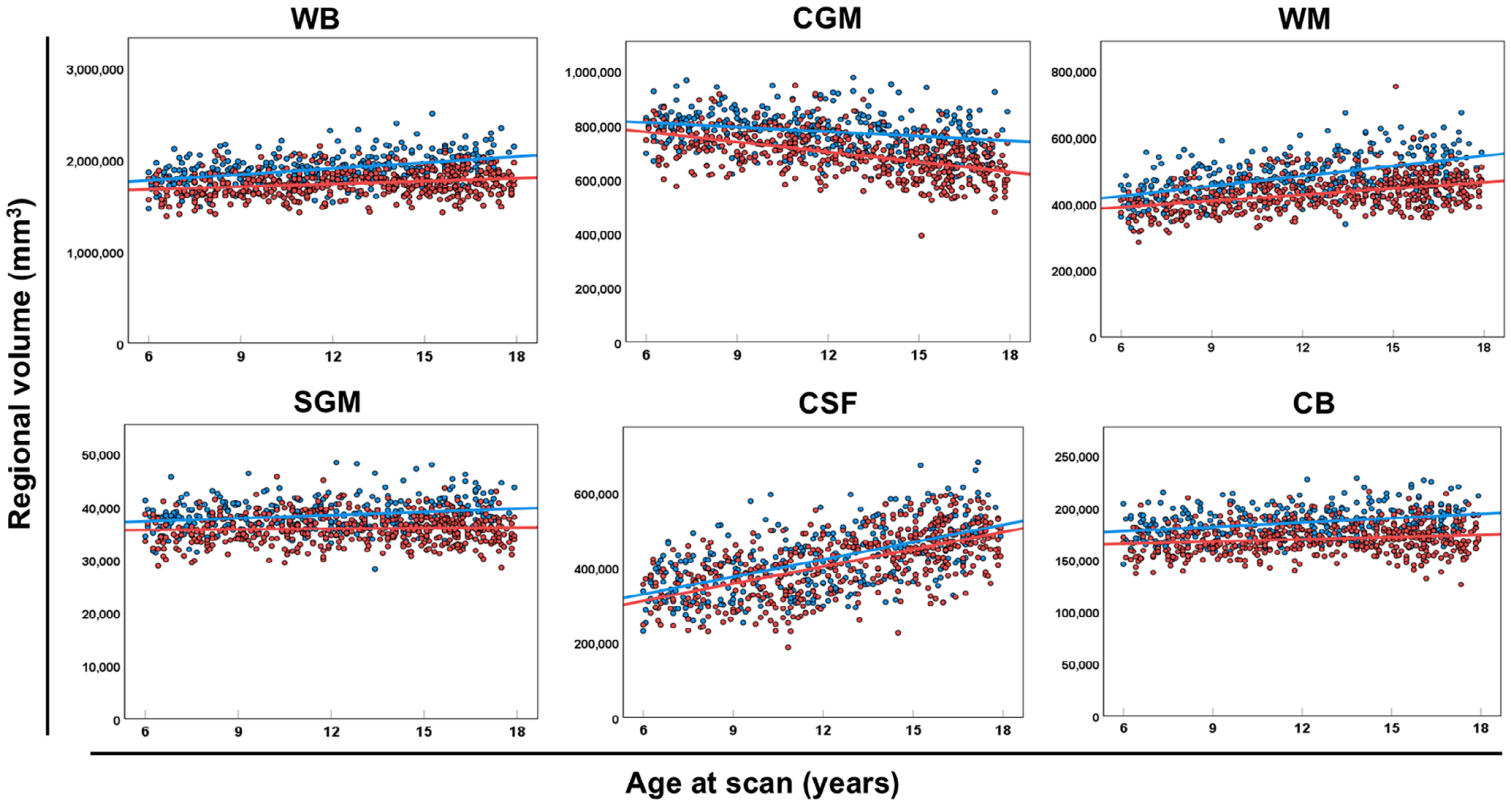
Global volume of each brain classification. Scatter plots and regression lines (between age at the scan and regional volume of WB, CGM, WM, SGM, CSF, and CB) in male (blue circles and lines) and female (red circles and lines) neurotypical controls. CB, cerebellum and brainstem; CGM, cerebral gray matter; CSF, extra‐axial cerebrospinal fluid; SGM, subcortical gray matter and fornix; WB, whole brain; WM, white matter. Figure reproduced from Shiohama et al. *Diagnostics* (2023) [65], licensed under CC BY 4.0.

Statistical harmonization requires that imaging conditions be standardized as much as possible. Accordingly, large‐scale brain MRI studies, such as the Adolescent Brain Cognitive Development (ABCD) Study [61] for children, the Human Connectome Project (HCP) [62] for adults, and the UK Biobank [63], provide vendor‐specific standard sequences. Once harmonization is achieved, individual brain metrics can be mapped onto age‐ and sex‐specific growth curves to yield clinically meaningful percentiles or *z*‐scores. Reference curves have been established for volume, cortical thickness, and surface area across pediatric populations [53, 60, 64, 65]. Our study provided cross‐site normative reference values of global and regional brain volumes by sex and age in children and adolescents (Figure 2) [65], based on MRI examinations of 846 neurotypical participants aged 6.0–17.9 years using region‐based analysis with the CIVET 2.1.0. pipeline [9] and ComBat‐GAM harmonization [58, 59].

Although harmonization techniques and normative reference curves enable the transformation of individual morphometric measures into standardized scores, caution is warranted when interpreting these metrics at the single‐subject level. Most quantitative MRI findings are derived from group‐level statistical comparisons, and their direct application to individual diagnosis remains limited [66]. Even when z‐scores or centile rankings are available, measurement variability, residual scanner effects, and biological heterogeneity can lead to substantial overlap between healthy and pathological populations [67]. As a result, false‐positive and false‐negative classifications may occur, potentially leading to inappropriate clinical interpretation or delayed diagnosis.

Furthermore, the reliability of individual‐level inference is influenced by test–retest variability and methodological differences across pipelines, which may approach or exceed the magnitude of disease‐related effects in some regions [43, 68]. Therefore, quantitative MRI metrics should be interpreted in conjunction with clinical findings and other biomarkers, rather than used as standalone diagnostic indicators. Continued efforts to establish robust individual‐level prediction models and validation in prospective clinical cohorts are essential for safe clinical translation.

## 4 Evaluation by the Diffusion‐Weighted MRI Tractography

Diffusion tensor imaging (DTI) has markedly enhanced the understanding of white matter microstructure and neural connectivity. DTI utilizes the anisotropic diffusion of water molecules in neural tissues to map the orientation and integrity of white matter tracts in vivo, offering critical insights into the structural organization of the human brain [69, 70]. Unlike conventional DWI, DTI requires multi‐directional (minimum 30 directions) *b* = 1000 s/mm^2^ acquisitions, along with additional DWI data acquired in reverse phase‐encoding to correct for eddy current–induced distortions.

The principal metric derived from DTI, fractional anisotropy (FA), quantifies the degree of directional diffusion and reflects microstructural properties such as axonal density and myelination. Complementary indices—mean diffusivity (MD), axial diffusivity (AD), and radial diffusivity (RD)—offer distinct insights into axonal integrity and demyelination processes [71, 72]. These indices are widely applied in both clinical and research contexts to evaluate brain development, aging, and a range of neurological and psychiatric disorders [73, 74, 75].

DTI has been instrumental in delineating brain developmental patterns. During childhood and adolescence, FA tends to increase while MD decreases, reflecting ongoing myelination and fiber organization [76, 77]. These trends have been consistently observed in both cross‐sectional and longitudinal studies, demonstrating the robustness of DTI in characterizing neurodevelopmental trajectories [78, 79]. Software tools such as TrackVis and DSI Studio enable the quantification of fiber characteristics, including length, volume, FA, and Apparent Diffusion Coefficient (ADC), through ROI‐based tractography [15, 16, 80]. FA typically increases postnatally due to axonal maturation, whereas ADC decreases as myelination and synaptic pruning progress [81, 82].

DTI serves as a sensitive biomarker for detecting microstructural abnormalities associated with various pathological conditions. In autism spectrum disorder (ASD), altered white matter connectivity and atypical FA values have been consistently reported, particularly in tracts related to social cognition [83, 84]. Similarly, studies of schizophrenia have identified disrupted frontotemporal connections and decreased FA in major association fibers [85, 86].

DTI also provides mechanistic insights into neurodegenerative diseases. In Alzheimer's disease, reduced FA and elevated MD values in the corpus callosum and cingulum bundle correlate with cognitive decline [87, 88]. Moreover, DTI metrics have proven useful in cases of mild traumatic brain injury (mTBI), where conventional imaging techniques often fail to detect subtle diffuse axonal injuries [89, 90].

Despite its strengths, DTI remains constrained by susceptibility to motion artifacts, partial volume effects, and simplified model assumptions that may not fully capture the complexity of neural fiber architecture [91]. Ongoing refinement of acquisition protocols and post‐processing algorithms is therefore essential to overcome these limitations and enhance clinical applicability [92, 93]. Compared with structural MRI, DTI indices are more vulnerable to inter‐scanner bias. Nonetheless, diffusion‐based reference curves for white matter integrity metrics (e.g., FA) across pediatric age groups have recently been established using ComBat‐GAM harmonization [94], similar to anatomical structure–based datasets.

An additional application of DTI is Diffusion Tensor Image Analysis along the Perivascular Space (DTI‐ALPS) method, which introduces post‐processing to assess the interstitial fluid‐cerebrospinal fluid (CSF) exchange mechanism—often referred to as the glymphatic system—in the human brain. This approach provides a noninvasive and indirect means of evaluating glymphatic function, and owing to its simplicity and clinical feasibility, it has been increasingly adopted in clinical research [95, 96].

**FIGURE 3:**
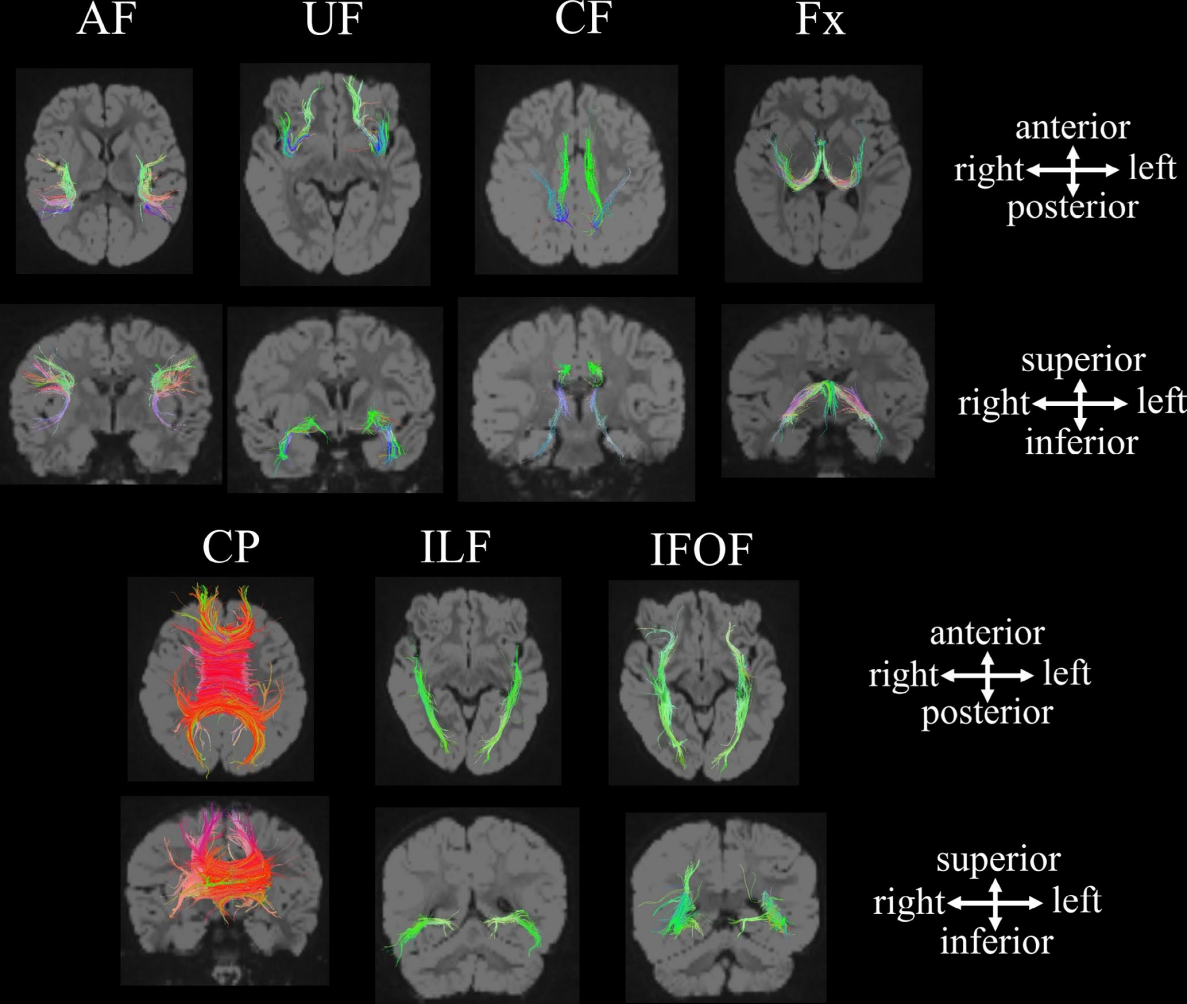
Ventral and front views of high‐angular resolution diffusion MR imaging tractography of a 2.5‐year‐old neurotypical girl (reproduced with permission to reuse [102]). AF, arcuate fasciculus; CF, cingulum fasciculus; CP, callosal pathway; Fx, fornix; IFOF, inferior fronto‐occipital fasciculus; ILF, inferior longitudinal fasciculus; UF, uncinate fasciculus.

Beyond DTI, several tractography algorithms have been developed, including fiber assignment by continuous tracking (FACT) [97], high‐angular resolution diffusion imaging (HARDI) [98], and constrained spherical deconvolution (CSD) [99]. Notably, HARDI and CSD have substantially improved the capacity to resolve complex fiber crossings, thereby broadening the diagnostic utility of diffusion imaging [100, 101]. These algorithms were initially implemented for ROI‐based tractography and enable visualization of white matter connectivity. We present high‐angular‐resolution diffusion imaging (HARDI) tractography‐derived fibers with ROI orientation in a typically developing female participant (Figure 3) [102].

In addition to ROI‐based approaches, advanced frameworks such as tract‐based spatial statistics (TBSS) [103], TRActs Constrained by UnderLying Anatomy (TRACULA) [104], MRtrix3 [105], and TractSeg [106] which operate on the AI platform PyTorch (https://pytorch.org/) have been introduced. These newer tractography techniques minimize registration errors, enhance reproducibility, and facilitate comprehensive assessments of white matter architecture.

As described above, diffusion‐weighted MRI tractography represents a pivotal advancement in neuroimaging, enabling the noninvasive exploration of white matter architecture and integrity. Its applications in developmental neuroscience, clinical diagnostics, and brain connectivity research underscore its enduring relevance and potential for continued innovation.

## 5 Clinical Translation and Integration

The integration of pediatric Quantitative magnetic resonance imaging (qMRI) into clinical workflows requires methodological rigor and standardization. Data must first be harmonized to minimize scanner‐dependent artifacts, followed by the transformation of individual morphometric and diffusion metrics into centile‐based normative scores. Once harmonized and benchmarked, disorder‐specific metrics can then be systematically interpreted in a patient‐centered context. The resulting reports include both absolute measurements and percentile deviations, enabling clinicians to monitor longitudinal trajectories and support individualized clinical decisions.

To clarify disease‐specific metrics, we performed brain MRI in patients with a range of conditions, from congenital genetic disorders (e.g., Rett syndrome, Down syndrome, CHARGE syndrome, macrocephalic disorders) to neurodevelopmental disorders (e.g., ASD, attention‐deficit/hyperactivity disorder, and Tourette syndrome).

ASD represents one of the most extensively studied applications of pediatric qMRI analysis, particularly for early risk stratification. However, several early intervention strategies have been proposed; only 42%–50% of children receive an ASD diagnosis by age 3 [107, 108]. This gap highlights the need for early diagnostic approaches beyond psychometric assessments. To identify imaging biomarkers for the early diagnosis of ASD, multiple studies have investigated brain MRI in infants who later develop the condition.

Our study retrospectively analyzed brain MRI metrics of 85 patients diagnosed with ASD after 3 years of age and compared them with those of 45 age‐ and sex‐matched non‐ASD controls. Patients with congenital anomaly syndromes, epilepsy, or intellectual disabilities were excluded. Multivariate analysis controlling for age, sex, and preterm birth revealed reduced nucleus accumbens volume and enlarged ventricles (lateral, third, and fourth) in the ASD group [109]. Enlargement of the lateral ventricles has also been reported in adults with ASD [110]. Consistent with previous studies [111, 112, 113], no significant differences were observed in the total volumes of the cerebral cortex, white matter, or basal ganglia [109]. We conclude that the volumes of the nucleus accumbens and the ventricular system may serve as imaging biomarkers predictive of ASD onset. Although autism has traditionally been associated with macrocephaly, brain parenchyma volume does not appear to increase in affected individuals, excluding those with genetic syndromes. While the relationship between ventricular enlargement and ASD remains unclear, population‐based cohort studies have reported a potential association between ASD and hydrocephalus [114]. Moreover, because the nucleus accumbens is a target site for oxytocin—a neuropeptide that has demonstrated efficacy in improving social functioning in ASD [115, 116]—its volume may represent a promising therapeutic biomarker for this disorder.

A longitudinal study conducted by other researchers found that amygdalar overgrowth emerges between 6 and 12 months of age in infants who later develop ASD, with accelerated growth predicting poorer social outcomes at 24 months [117]. Complementary evidence indicates that enlarged perivascular spaces and increased extra‐axial cerebrospinal fluid volumes become detectable between 12 and 24 months, suggesting early disruptions in glymphatic clearance pathways [118]. Functional MRI studies have also revealed atypical amygdala–visual cortex connectivity during the first year of life, reflecting early alterations in neural circuitry [119]. Differences in results across studies may partly reflect variations in design—such as prospective versus retrospective approaches, differences in genetic risk, and the exclusion of infants with epilepsy and/or preterm birth. Most previous studies prospectively enrolled infants with both high and low genetic risks while excluding preterm infants.

## 6 Conclusion

Advancements in anatomical structure analysis and diffusion‐weighted MRI tractography have substantially improved the phenotyping of brain morphology. Although these methods are widely used in disease characterization and diagnostic biomarker discovery, challenges such as inter‐scanner bias continue to limit their clinical implementation. Establishing standardized protocols shared between clinical practice and research will be essential to support future multicenter studies. Importantly, careful consideration of statistical limitations in individual‐level inference and the development of unified longitudinal processing frameworks will be essential for translating quantitative MRI into reliable clinical tools.

## Author Contributions

T.S. designed the study, drafted the manuscript and figure, and approved the final manuscript.

## Funding

This study was funded by the Japan Society for the Promotion of Science (JSPS) KAKENHI (JP24K02392), Intramural Research Grant for Neurological and Psychiatric Disorders of the National Center of Neurology and Psychiatry (NCNP) (6‐10), and Kawano Masanori Memorial Public Interest Incorporated Foundation for Promotion of Pediatrics (36‐13).

## Conflicts of Interest

The author declares no conflicts of interest.

## Data Availability

The data that support the findings of this study are available from the corresponding author upon reasonable request.
